# Brachytherapy With Silicone Oil for Radiation Attenuation in the Surgical Management of Ciliary Body Leiomyoma

**DOI:** 10.7759/cureus.116902

**Published:** 2026-09-25

**Authors:** Tara A McCannel, Mathew B Krick, Ben J Glasgow

**Affiliations:** 1 Vitreoretinal Surgery Service, UCLA Jules Stein Eye Institute, Los Angeles, USA; 2 Ophthalmology, Boston University Chobanian & Avedisian School of Medicine, Boston, USA; 3 Ocular Oncology, UCLA Jules Stein Eye Institute, Los Angeles, USA; 4 Eye Pathology, UCLA Jules Stein Eye Institute, Los Angeles, USA

**Keywords:** brachytherapy, ciliary body tumor, leiomyoma, radiation attenuation, silicone oil, uveal melanoma, uveal tumor, vitrectomy

## Abstract

We report a case of ciliary body intraocular leiomyoma treated with brachytherapy and vitrectomy with silicone oil 1000 centistokes for radiation attenuation.

A review of the clinical course of a patient with treated ciliary body intraocular leiomyoma was performed, including patient demographics, baseline and final visual acuity, multimodal imaging results, tumor control, and surgical technique. Brachytherapy and vitrectomy with silicone oil for radiation attenuation resulted in regression of the intraocular leiomyoma, resolution of serous retinal detachment, and 20/25 final vision at six years.

Vitrectomy with silicone oil 1000 centistokes for radiation attenuation is a feasible technique for intraocular leiomyoma to achieve both tumor control and optimal vision results. This approach offers a vision-preserving alternative for intraocular tumors requiring radiotherapy.

## Introduction

Intraocular leiomyoma is a rare and benign smooth muscle tumor that typically affects the ciliary body and iris [1]. Vitrectomy is a surgical procedure involving removal of the vitreous humor from the eye and is often used in the surgical treatment of intraocular tumors [2]. Vitrectomies may improve intraocular tumor visualization, enabling surgeons to obtain more precise biopsies and surgical access to tumors, which subsequently allow for histopathological analysis and increased diagnostic accuracy [3].

In the context of intraocular tumor treatment, vitrectomies are often paired with plaque brachytherapy, which is a procedure used to irradiate tumors and consequently reduce tumor size. However, plaque brachytherapy may lead to unwanted complications, such as radiation retinopathy, resulting from radiation exposure to the eye. 

Replacement of vitreous humor with silicone oil 1000 centistokes after vitrectomy has previously been demonstrated to reduce radiation retinopathy and result in significantly improved visual acuity when performed with brachytherapy in patients with large uveal melanoma [4-6]. Specifically, replacement of vitreous humor with silicone oil after vitrectomy protects against radiation damage by allowing silicone oil to absorb radiation and therefore shield healthy retinal tissue from radiation damage. Here, we report a case of ciliary body intraocular leiomyoma treated with this technique and the outcomes at six years.

## Case presentation

A 46-year-old healthy Hispanic male, found to have a suspicious intraocular mass in the left eye, was referred to the Ocular Oncology Center, Jules Stein Eye Institute, UCLA. He had been experiencing gradually decreasing vision in the left eye for the past two months. 

Visual acuity was 20/40 in the right eye and 20/150 in the left eye. Intraocular pressures were 14 mmHg in the right eye and 16 mmHg in the left eye. Anterior segment examination revealed a focal posterior subcapsular cataract superior nasally (Figure 1) in the left eye. Dilated fundus examination of the left eye revealed an elevated smooth-shaped pigmented ciliochoroidal mass in the superior nasal fundus and inferior serous retinal detachment. Examination of the right eye was normal. 

**Figure 1:**
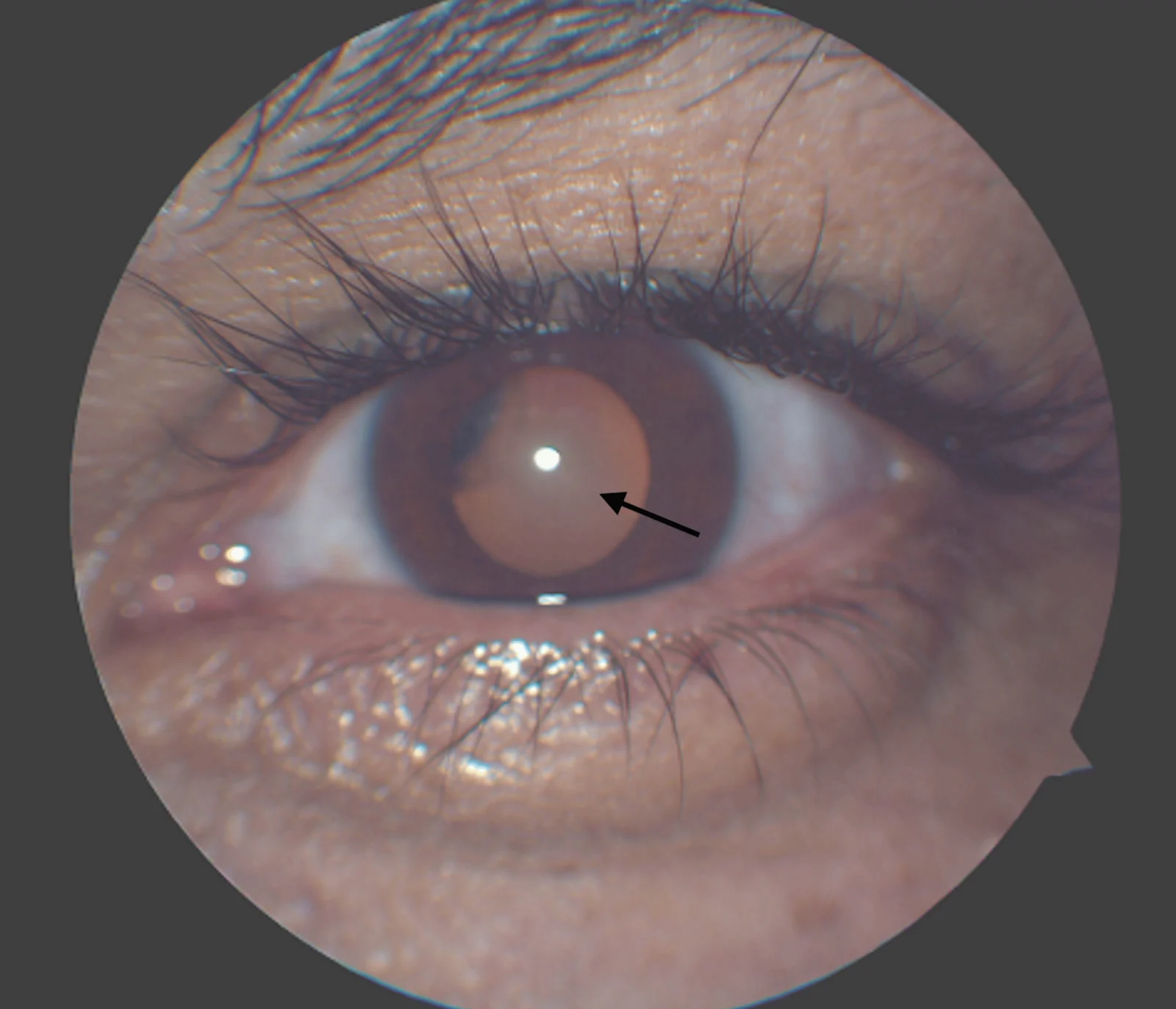
External photograph demonstrating focal nasal cataract against red reflex in the left eye

Optos wide-angle color imaging (Optos plc., Dunfermline, Scotland, United Kingdom) (Figure 2A) and fluorescein angiography (Figure 2B) revealed staining at the base of the mass, as well as mild vascular leakage inferiorly at the retinal detachment. Ultrasonography revealed a dome-shaped ciliary body and choroidal lesion of height 8.04 mm, with greatest basal diameter 12.62 mm (Figure 3). Extensive inferior serous retinal detachment was also shown by ultrasound. Immersion ultrasound biomicroscopy indicated notable ciliary body involvement (Figure 4). A-scan ultrasonography revealed low to medium reflectivities with prominent vascularity present. PET-CT scan was performed with negative findings elsewhere.

**Figure 2:**
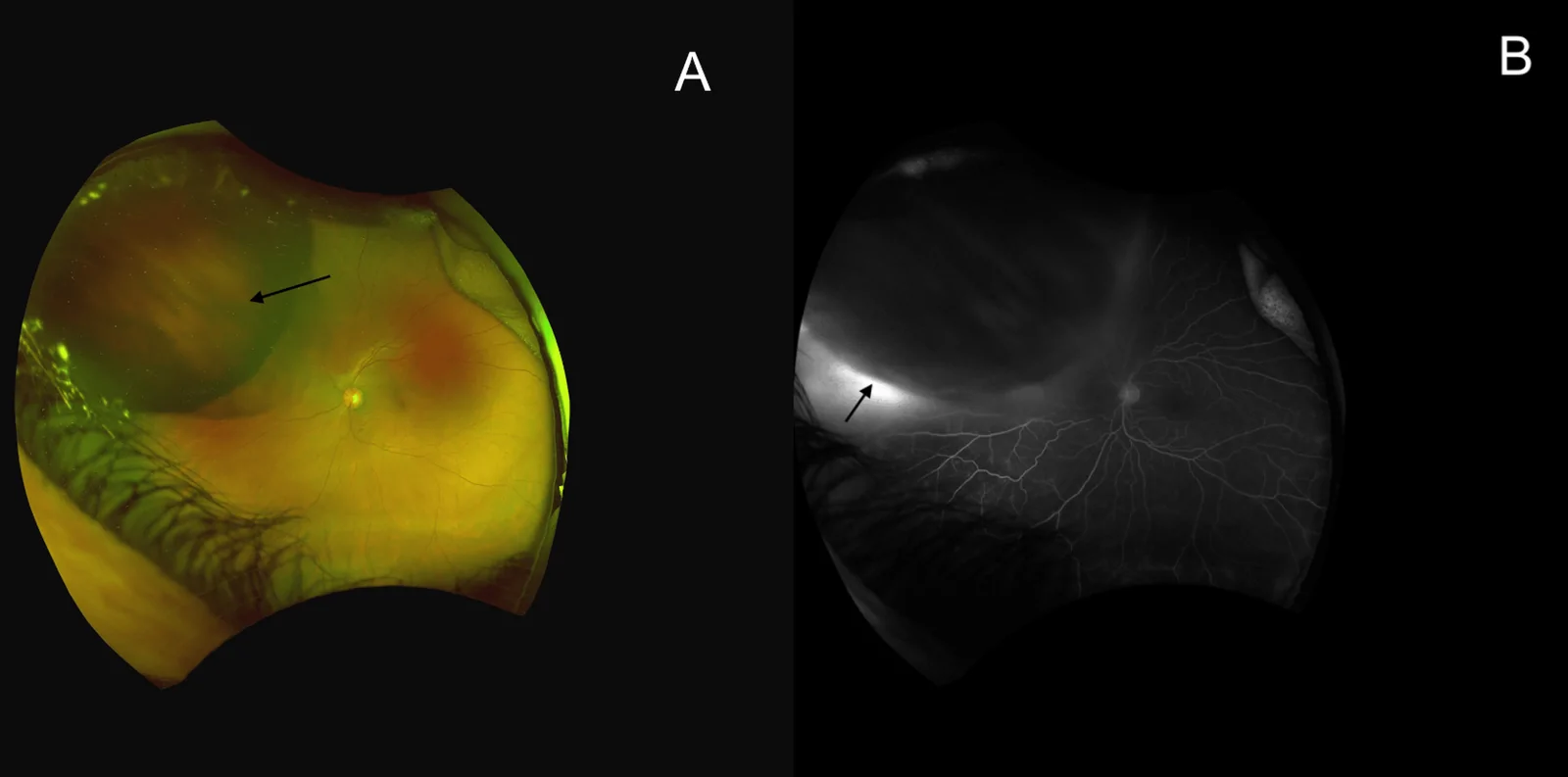
Visualization of ciliary body mass with Optos wide-angle color photograph and Optos wide-angle fluorescein angiogram Figure 2A shows an Optos wide-angle color photograph demonstrating a pigmented ciliochoroidal mass lesion (black arrow) with inferior retinal detachment in the left eye. Figure 2B shows an Optos wide-angle fluorescein angiogram, late phase, demonstrating staining at the base of the mass (black arrow) and faint leakage inferiorly corresponding to retinal detachment in the left eye. Optos retinal imaging by Optos plc., Dunfermline, Scotland, United Kingdom.

**Figure 3:**
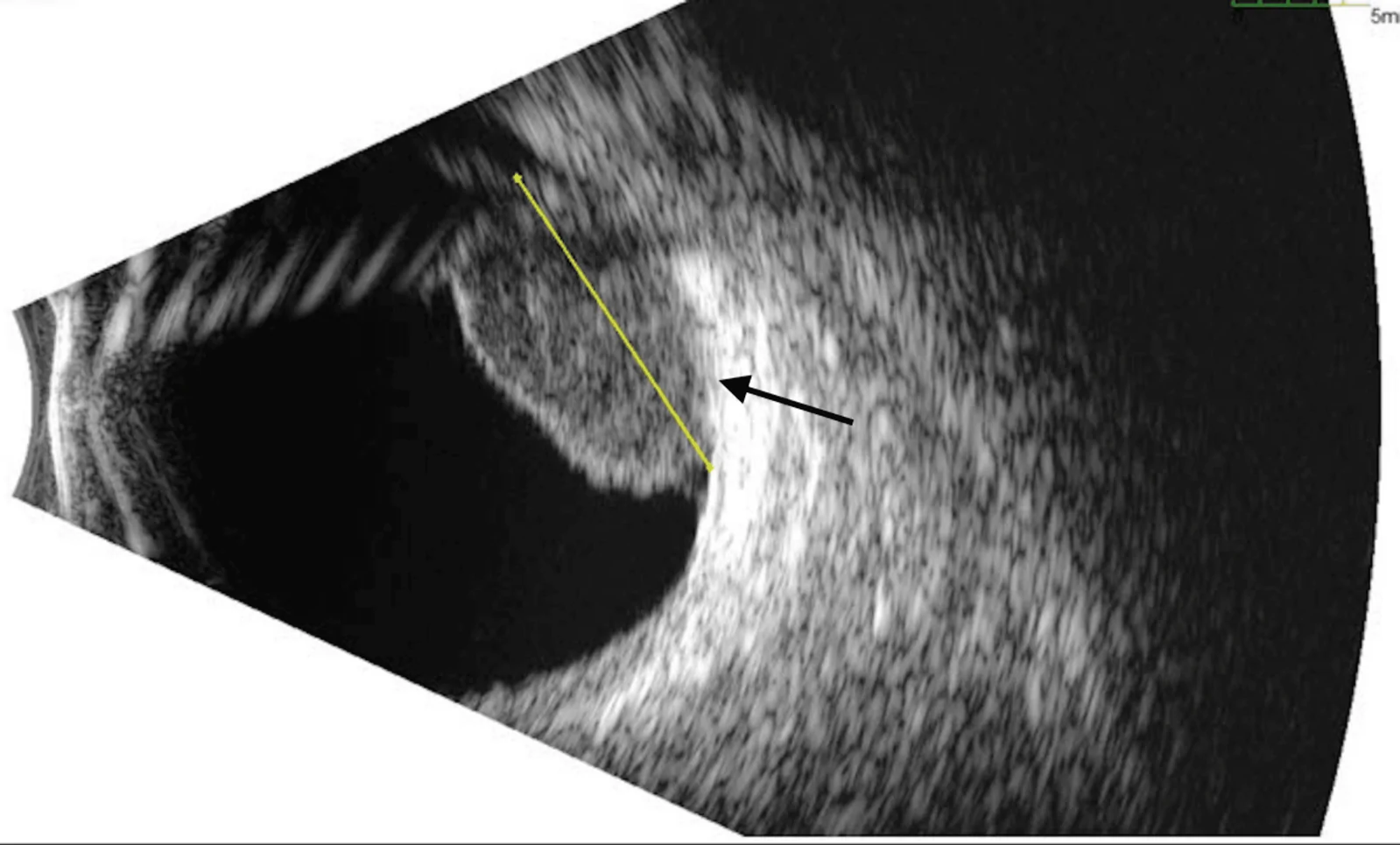
B-scan ultrasonography of the ciliary body mass B-scan ultrasonography reveals a dome-shaped mass lesion (black arrow) with a thickness of 8.04 mm (yellow caliper).

**Figure 4:**
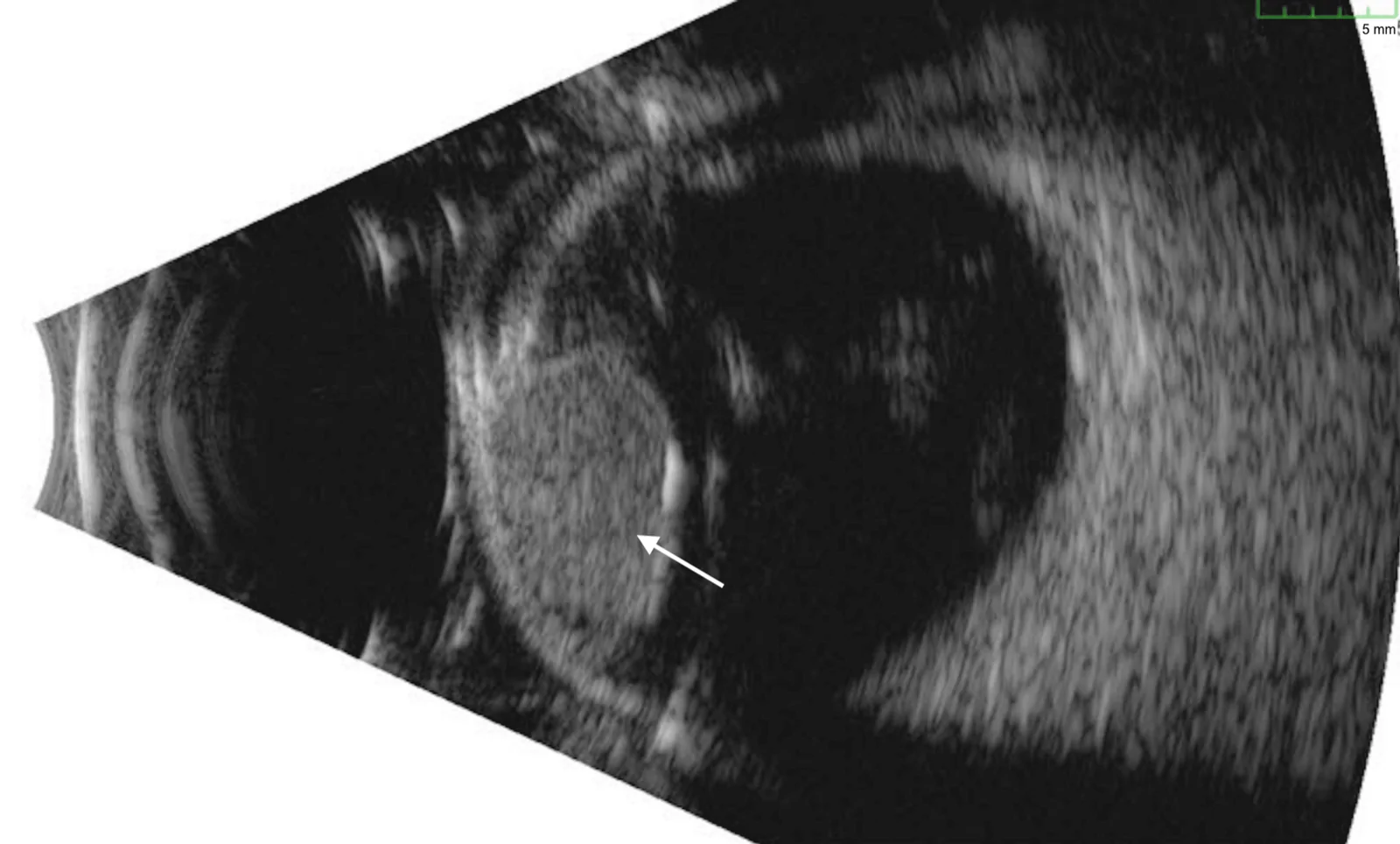
Ultrasound biomicroscopy demonstrating ciliary body location of the mass lesion

The preliminary diagnosis was ciliochoroidal melanoma. To prevent radiation retinopathy, radiation attenuation was performed with silicone oil 1000 centistokes as a vitreous attenuating substance [4-6]. The surgical procedure performed was as follows: phacoemulsification with intraocular lens placement, transscleral fine-needle aspiration biopsy, ultrasound-guided Iodine-25 plaque brachytherapy with a total activity of 54.39 mCi, pars plana vitrectomy, air-fluid exchange, and placement of silicone oil 1000 centistokes.

Prognostic fine-needle biopsy for gene expression profiling using the Castle Biosciences DecisionDx test (Castle Biosciences, Friendswood, USA) revealed a Class 1A signature, with a negative *PRAME *(preferentially expressed antigen in melanoma)** **mutation. Cytogenetic fine-needle biopsy testing for aberrations in chromosomes 3 and 6 was negative. Anti-*BRCA1*-associated protein 1 (anti-*BAP1*)** **immunohistochemical staining showed nuclear reactivity, suggesting normal expression of BAP1 protein [7].

Fine-needle biopsy submitted to pathology revealed spindle cells, non-pigmented, in a neurofibrillary background. Anti-melanoma antigen recognized by T cells 1 (anti-MART1) was non-reactive in tumor cells (Figure 5). Anti-smooth muscle actin (anti-SMA) was reactive in the cytoplasm of spindle cells, consistent with the diagnosis of ciliary body leiomyoma (Figure 5B).

**Figure 5:**
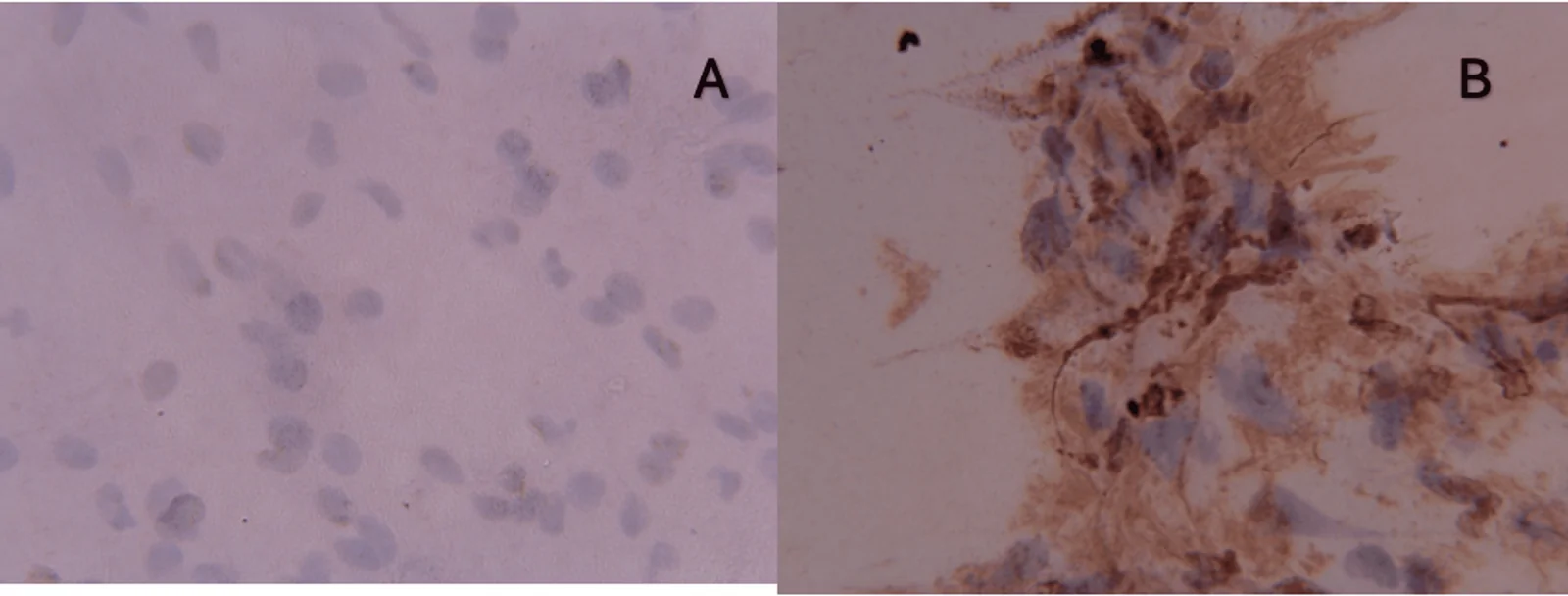
Fine-needle aspiration smears A. Anti-melanoma antigen recognized by T cells 1 (anti-MART-1) antibody is non-reactive.  B.  Anti-smooth muscle actin (anti-SMA) is reactive in the cytoplasm of all cells and is strongly concentrated in the dendritic extensions of the spindle cells.

Six years after treatment, following the removal of silicone oil at 6 months post-operatively, the patient’s vision in the treated left eye was 20/25.

Clinical evaluation revealed a well-positioned posterior chamber intraocular lens and a completely attached retina. The superonasal area on Optos wide-angle color imaging (Figure 6) revealed residual retino-choroidal atrophic changes in the location of the treated tumor. At five and a half years following plaque insertion, ultrasound biomicroscopy revealed a treated tumor thickness in the ciliary body region of 1.98 mm, compared to the baseline thickness of 8.04 mm (Figure 7). Macular optical coherence tomography (OCT) revealed a normal-appearing foveal contour with the absence of radiation macular edema (Figure 8). Visual field examination results remained stable.

**Figure 6:**
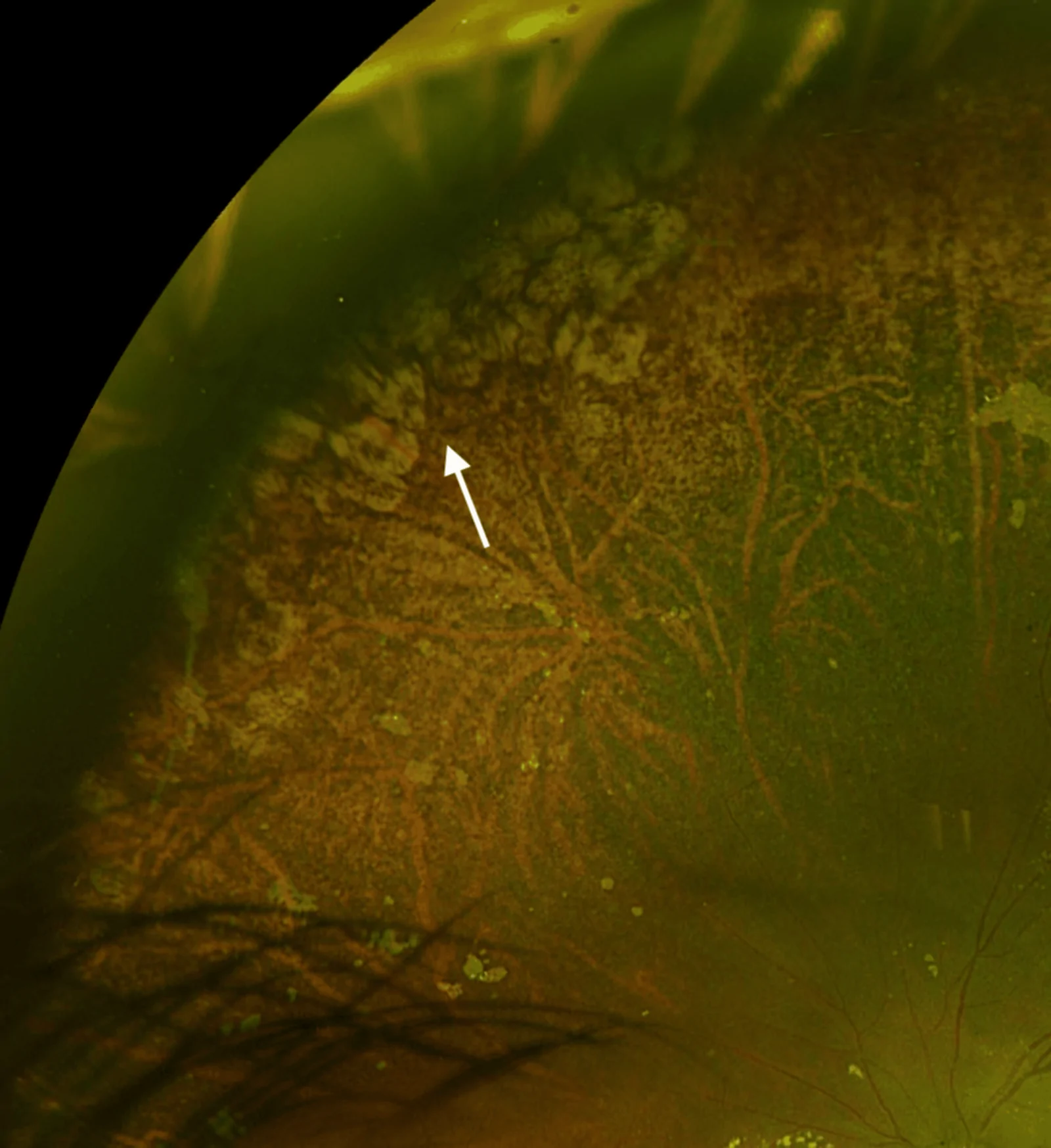
Optos photograph of treated ciliary body leiomyoma Six years after treatment, the superior nasal aspect of the Optos color photograph of the left eye demonstrates retinochoroidal atrophic changes at the location of the treated leiomyoma. Optos retinal imaging by Optos plc., Dunfermline, Scotland, United Kingdom.

**Figure 7:**
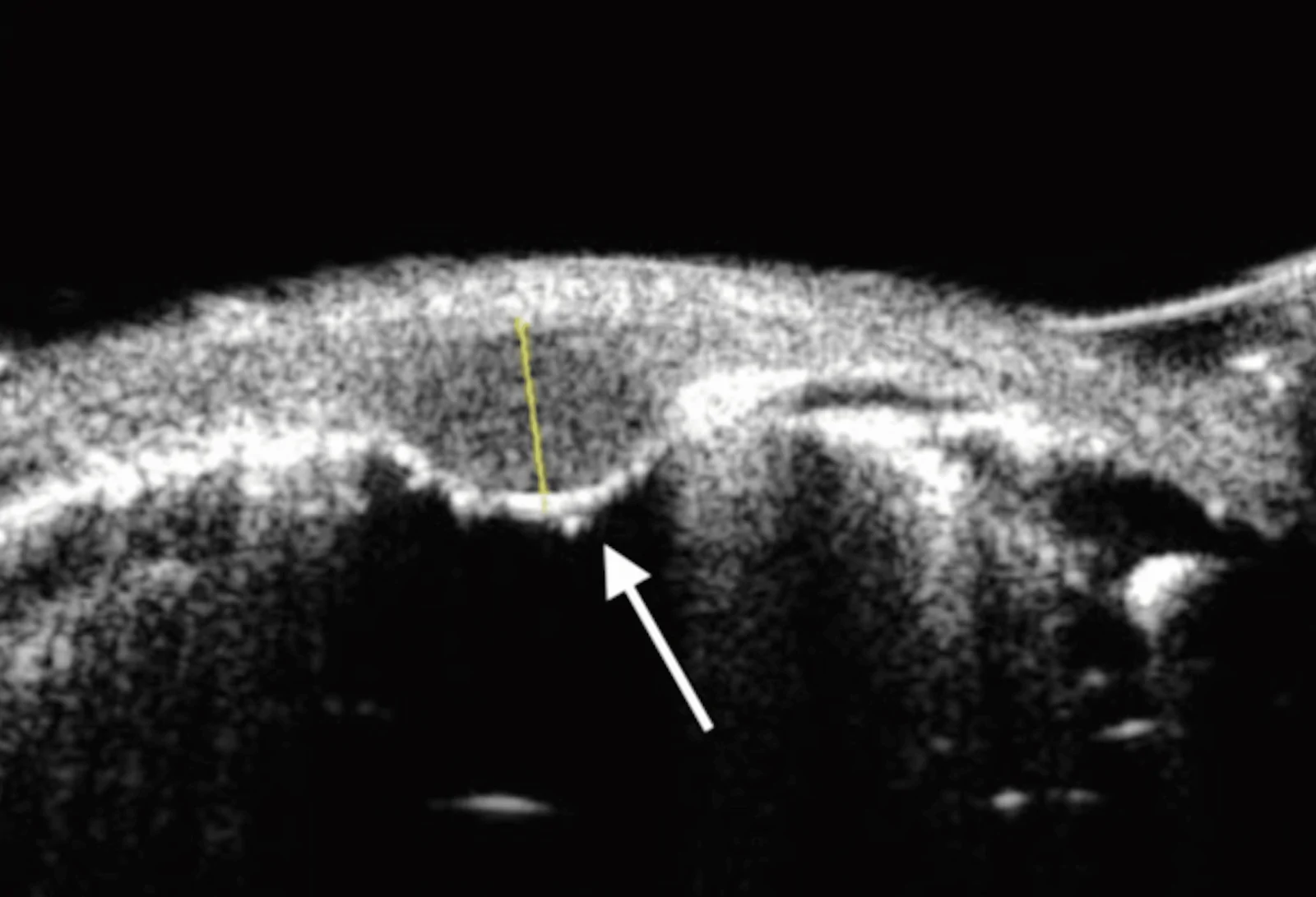
Ultrasound image of treated leiomyoma Ultrasound biomicroscopy demonstrating reduction in the thickness of the treated ciliary body leiomyoma to 1.98 mm (yellow caliper) after six years of follow-up.

**Figure 8:**
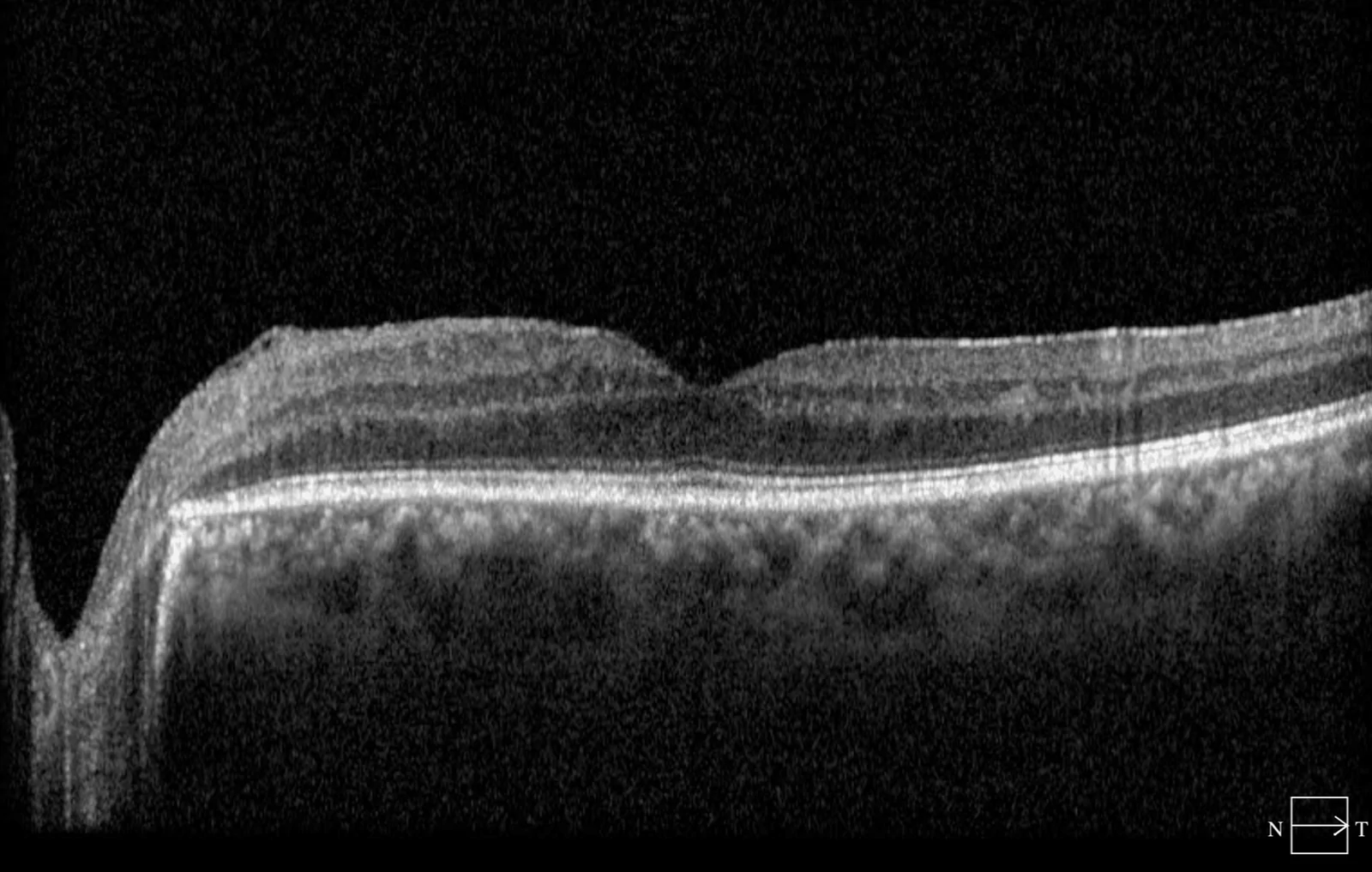
Optical coherence tomography (OCT) of treated ciliary body leiomyoma Optical Coherence Tomography (OCT) through the macula demonstrates a normal foveal contour and the absence of macular edema from radiation retinopathy six years after brachytherapy treatment with silicone oil radiation attenuation. Vision is 20/25.

## Discussion

Mesectodermal leiomyoma is a rare intraocular tumor. We report a case with a 6-year follow-up, in which a ciliochoroidal leiomyoma was successfully treated by brachytherapy and silicone oil for radiation attenuation, with excellent vision. Tomar et al. [1] published a review of the only 80 reported cases of intraocular leiomyoma from 1937 to 2019. Men represented 36% of these cases, and women, 64%. The mean age was 35.5 years (ranging from 8 to 80 years). The authors found that 40% of pigmented leiomyomas involved the ciliary body. Interestingly, pigmented leiomyomas may appear clinically identical to uveal melanoma. The use of anti-SMA staining on tumor sampling was the most reliable diagnostic tool when positive. A recent report of two clinically nearly identical cases published by Tomar et al. [8] described one patient with what turned out to be leiomyoma after enucleation, and one diagnosed as uveal melanoma after biopsy. 

The diagnosis of mesectodermal leiomyoma by fine-needle aspiration has been made in only two prior reports. One case was reported from our institution [9] and was confirmed by histopathology. A second case was reported by Kiratli et al. [10] and was similar to the current case in that the diagnosis was made exclusively by fine-needle aspiration. A common feature for the diagnosis is the neurofibrillary background with dendritic cytoplasmic features (Figure 6).

The management for intraocular leiomyoma is highly variable. Irradiation of intraocular leiomyomas, as with any intraocular tumor, may damage sensitive tissues and structures both within and around the eye, leading to greater risks of radiation complications such as radiation retinopathy, neovascular glaucoma, retinal detachment, and cataract [1,7,11]. In the review paper by Tomar et al. [1], it was found that 47.5% of the cases of leiomyoma were also managed with enucleation. Tumor excision represented 40.7% of management, and only 3.4% (two cases in the series) underwent radiation, with one of the two cases progressing to enucleation from neovascular glaucoma. Observation was reported as a management strategy in just 3.4% of the cases in this review.

The use of brachytherapy and radiation in general for the treatment of ocular tumors is indeed associated with visual loss due to radiation retinopathy. However, Silicone oil 1000 centistokes has been demonstrated by our group to be a radiation-attenuating substance for Iodine-125 when used as a vitreous substitute during plaque brachytherapy in the primary treatment of uveal melanoma [4-6]. In large uveal melanoma, we reported that using silicone oil to prevent radiation damage to the healthy tissues was found to significantly improve visual prognosis compared to brachytherapy alone [6]. 

In our case of intraocular leiomyoma, the technique of brachytherapy with vitrectomy and silicone oil for radiation attenuation resulted in a favorable tumor response and excellent vision at six years. Despite the non-malignant nature of leiomyoma, tumor growth with exudative retinal detachment may necessitate intervention for local tumor control and optimization of vision in these eyes. When there is visual potential, we feel that enucleation for these benign tumors must be avoided.

## Conclusions

We report a case of a left eye intraocular ciliary body leiomyoma, initially suspected to be a uveal melanoma, which was treated with plaque brachytherapy and vitrectomy with silicone oil for radiation attenuation and followed up over six years. Tumor control and optimal vision results were attained. The role of silicone oil as a vitreous attenuating substance to reduce radiation exposure should be further characterized in order to attain a better understanding of whether a protective association is present.
